# Uterine prostaglandin DP receptor-induced upon implantation contributes to decidualization together with EP4 receptor

**DOI:** 10.1016/j.jlr.2024.100636

**Published:** 2024-08-31

**Authors:** Risa Sakamoto, Takuji Fujiwara, Yuko Kawano, Shizu Aikawa, Tomoaki Inazumi, On Nakayama, Yukiko Kawasaki-Shirata, Miho Hashimoto-Iwasaki, Toshiko Sugimoto, Soken Tsuchiya, Satohiro Nakao, Toru Takeo, Yasushi Hirota, Yukihiko Sugimoto

**Affiliations:** 1 Department of Pharmaceutical Biochemistry, Graduate School of Pharmaceutical Sciences, Kumamoto University, Kumamoto, Japan; 2 Department of Physiological Chemistry, Graduate School of Pharmaceutical Sciences, Kyoto University, Kyoto, Japan; 3 Department of Obstetrics and Gynecology, Graduate School of Medicine, The University of Tokyo, Tokyo, Japan; 4 Division of Reproductive Engineering, Center for Animal Resources and Development, Kumamoto University, Kumamoto, Japan

**Keywords:** prostanoid receptors, stroma, decidua, gene expression, mouse, uterus, implantation, decidualization, eicosanoid, lipid mediator

## Abstract

To investigate the yet-unknown roles of prostaglandins (PGs) in the uterus, we analyzed the expression of various PG receptors in the uterus. We found that three types of Gs-coupled PG receptors, DP, EP2, and EP4, were expressed in luminal epithelial cells from the peri-implantation period to late pregnancy. DP expression was also induced in stromal cells within the mesometrial region, whereas EP4 was expressed in stromal cells within the anti-mesometrial region during the peri-implantation period. The timing of DP induction after embryo attachment correlated well with that of cyclooxygenase-2 (COX-2); however, COX-2-expressing stromal cells were located in the vicinity of the embryo, whereas DP-expressing stromal cells surrounded these cells on the mesometrial side. Specific [^3^H]PGD_2_-binding activity was detected in the decidua of uteri, with PGD_2_ synthesis comparable to that of PGE_2_ detected in the uteri during the peri-implantation period. Administration of the COX-2-specific inhibitor celecoxib caused adverse effects on decidualization, as demonstrated by the attenuated weight of the implantation sites, which was recovered by the simultaneous administration of a DP agonist. Such a rescuing effect of the DP agonist was mimicked by an EP4 agonist, but not an EP2 agonist. While the importance of DP signaling was shown pharmacologically, DP/EP2 double deficiency did not affect implantation and decidualization, suggesting the contribution of EP4 to these processes. Indeed, administration of an EP4 antagonist substantially affected decidualization in DP/EP2-deficient mice. These results suggest that COX-2-derived PGD_2_ and PGE_2_ contribute to decidualization via a coordinated pathway of DP and EP4 receptors.

Embryo implantation is elicited by a direct interaction between embryo and uterine tissues and is the key process for the establishment of pregnancy (1, 2). Upon attachment of blastocysts to the uterine epithelia, the surrounding stromal cells begin to differentiate into decidual cells, and trophoblast cells invade the deciduae by eliminating the epithelia, promoting formation of the placenta. Decidualization, which is the differentiation of stromal cells, accompanies unique morphological changes in tissue, including luminal epithelium (LE) breakdown and angiogenesis (3, 4, 5, 6, 7). Impaired decidualization leads to infertility, miscarriage, and preterm parturition (8). Despite the importance of decidualization in pregnancy, the molecular mechanisms underlying implantation and decidualization have remained unclear to date.

Prostaglandins (PGs) are biologically active lipids synthesized from arachidonic acid (AA), which is cleaved from membrane phospholipids by phospholipase (PL) A_2_ (9). PGs are synthesized by the action of cyclooxygenase (COX) and terminal PG synthases and then rapidly released extracellularly without being stored, exerting a wide variety of biological actions via the eight specific G protein-coupled receptors expressed in neighboring cells (10, 11). COX-1 is constitutively expressed to produce PGs that have housekeeping functions, whereas COX-2 is induced in response to proinflammatory and hormonal stimuli and produces PGs that have transient but strong effects (12, 13). It has been reported that COX-2-deficient mice show abnormalities in the processes of ovulation, fertilization, implantation, and decidualization, indicating that COX-2-derived PGs play roles in these early reproductive processes (14). Among them, implantation and decidualization are controlled by COX-2 and its PG products. Indeed, a previous report showed that COX-2 is induced in embryo-attached LE cells and stromal cells immediately below them (15). Furthermore, it has been reported that mice deficient in cytosolic phospholipase A_2_ α (cPLA_2_α; encoded by the *Pla2g4a* gene), which is usually responsible for supplying AA to COX-2, show similar implantation defects (16), also supporting the notion that COX-2-derived PGs play a pivotal role in the uteri of mice during early pregnancy. It was recently demonstrated that such induction of COX-2 expression is driven by the lysophosphatidic acid (LPA)-LPAR3 receptor axis originating in blastocysts and also that COX-2-derived PG triggers the decidualization of stromal cells (17, 18). An *in vitro* system using human endometrial stromal cells also showed that decidualization is stimulated by progesterone in a PG-cAMP-dependent manner (19). Nevertheless, it remains unclear as to which PG receptor signals contribute to the decidualization of stromal cells.

Eight PG receptors have been shown to form unique subfamilies depending on the signaling they are coupled to, suggesting that PG receptors have evolved in a signal-dependent manner (20). Type-D prostanoid receptor (DP; the same applies to all receptor abbreviations hereafter), EP2, EP4, and IP are coupled to the cAMP formation (stimulatory G protein [Gs]), EP3 is coupled to the inhibition of adenylyl cyclase (Gi), and EP1, FP, and TP are coupled to the stimulation of phospholipase C (Gq). Thus, PG receptors coupling to the same signaling may share several functions; for example, the Gs-coupled PGE receptors EP2 and EP4 are coexpressed on immune cells, such as macrophages and T-cells, and exert similar effects (21, 22, 23). It has also been reported that in the uterus, PG receptors are expressed in the myometrium, LE, and stromal cells, with unique spatiotemporal expression patterns (24, 25). Therefore, it is assumed that Gs-coupled PG receptors compensate for each other in the stimulation of stromal cells, and that the single deficiency of each receptor alone does not impair decidualization (26). Presumably owing to these reasons, it remains unclear as to which Gs-coupled PG receptors are involved in implantation and decidualization.

In this study, to gain insight into the PG receptors involved in reproductive processes, including implantation and decidualization, we screened for PG receptors expressed in uterine tissues, particularly focusing on the Gs-coupled receptors DP, EP2, and EP4.

## Materials and Methods

### Mice and tissue preparation

Female C57BL/6 mice were purchased from Japan SLC (Hamamatsu) and maintained at 23 ˚C under a 12 h light/dark cycle. To obtain timed-pregnant mice, C57BL/6 virgin female mice (8–12 weeks of age) were housed overnight with C57BL/6 male mice, and checked the following morning for vaginal plugs. The day when a vaginal plug was observed was counted as day 0.5 post-conception. Uteri were isolated from nonpregnant mice or mice at various gestational days, buried in Optimal Cutting Temperature Compound (Sakura Finetek Japan), frozen, and then sectioned at 10-μm thickness, and cryopreserved for *in situ* hybridization analysis. Alternatively, uteri were quickly frozen in liquid nitrogen, and stored in a deep freezer for RNA isolation and subsequent conventional and real-time reverse transcription-polymerase chain reaction (RT-PCR) analysis. All animal experiments were conducted using procedures approved by the Experimental Animal Ethics Committee of Kumamoto University (Kumamoto).

### Conventional and real-time RT-PCR analysis

Total RNA was extracted from uteri using Sepasol-RNA I Super G (Nacalai Tesque) and reverse transcription was performed using PrimeScript RT Reagent Kit (Takara Bio). PCR was performed using specific primers and Fast SYBR Green Master Mix (Applied Biosystems). The expression level of each gene was corrected with the β-actin expression value of each sample. Primer pairs used in this study are listed in supplemental Table S1.

### In situ hybridization

In situ hybridization was carried out as described previously (27). Uterine horns were dissected and immediately frozen in liquid nitrogen. Sections (10 μm in thickness) were cut on a Jung Frigocut 3000E cryostat (Leica Instruments) and thaw-mounted onto poly-L-lysine-coated glass slides. Mouse cDNAs for DP (28), EP2, EP4 (25), and COX-2 (29) were prepared in the pBluescript II vector (Stratagene), and antisense or sense riboprobes specific for each transcript were synthesized by transcription with T3 or T7 RNA polymerase (Stratagene) in the presence of [α-^35^S]CTP. The sections were fixed with 4% formalin and acetylated with 0.25% acetic anhydride. Hybridization was carried out in a buffer containing 50% formamide, 2 × saline-sodium citrate (SSC; 0.3 M NaCl, 30 mM trisodium citrate [pH 7.0]), 10 mM tris(hydroxymethyl)aminomethane-Cl (pH 7.5), 1 × Denhardt’s solution, 10% dextran sulfate, 0.2% sodium dodecyl sulfate, 100 mM dithiothreitol, 500 μg/ml sheared single-stranded salmon sperm DNA, and 250 μg/ml yeast tRNA. The antisense riboprobes were added to the hybridization buffer at 1.5 × 10^5^ cpm/μl. After incubation at 60˚C for 5 h, the slides were washed for 1 h in 2 × SSC. The sections were treated with 20 μg/ml ribonuclease A, followed by an additional wash in 0.1 × SSC at 60°C for 1 h. The slides were then dipped in nuclear track emulsion (NTB3, Eastman Kodak). After exposure for 4 weeks at 4°C, the dipped slides were developed, fixed, and counter-stained with hematoxylin and eosin. Alternatively, in situ hybridization was performed using digoxygenin (DIG)-labeled probes, as previously reported (30). After the hybridization procedure, DIG probes were detected by an alkaline phosphatase anti-DIG antibody and 5-bromo-4-chloro-3-indolyl- phosphate/nitro blue tetrazolium color development substrate (Promega), with counterstaining of nuclei by Kernechtrot (Muto Pure Chemicals). These experiments were repeated two or three times with different mice and similar results were obtained.

### [^3^H]PGD_2_ and [^3^H]PGE_2_ binding assay

Uterine horns of pregnant mice on day 7.5 were dissected, freed from the fetuses, and their endometrial tissues were stripped from myometria and immediately frozen. Endometria were homogenized with a Potter-Elvehjem homogenizer in 10 mM 2-morpholinoethanesulfonic acid/NaOH, (pH 6.0), containing 10 mM MgCl_2_, 1 mM ethylenediaminetetraacetic acid, 20 μM indomethacin and 0.1 mM phenylmethylsulfonyl fluoride. After centrifugation of the homogenate at 250,000 *g* for 10 min, the pellet was washed and suspended in the same buffer. The membrane fraction (200 μg) was incubated with 40 nM of [^3^H]PGD_2_ at 4°C for 1 h, and [^3^H]PGD_2_ bound to the membrane fraction was determined by adding a 1,000-fold excess of unlabeled PGD_2_ to the incubation mixture. Specific binding was calculated by subtracting the non-specific binding from the total binding. Specific [^3^H]PGE_2_ binding was also determined in the same manner.

### Measurement of PGs in uterine implantation sites

Implantation sites were isolated from pregnant mouse uteri, and lipid extraction and quantitative analysis of lipids were performed as previously described (31). Briefly, frozen tissues were homogenized in methanol using a beads homogenizer. Lipids were then purified by solid-phase extraction using Oasis hydrophilic lipophilic balance extraction cartridges (Waters), with the deuterium-labeled internal standard PGE_2_-d4. High-performance liquid chromatography-negative-ion electrospray ionization-tandem mass spectrometry analyses were performed using Nexera X2 (Shimadzu) and the triple quadruple mass spectrometer QTRAP 5500 (SCIEX). A reverse-phase column (Kinetex C18, 1.7 μm, 150 mm × 2.1 mm, Phenomenex) was used for chromatographic separation. Samples were eluted using mobile phase A (0.1% formic acid in water) and B (acetonitrile). The amounts of AA and PGs were quantified by multiple reaction monitoring, and the transitions for AA, PGE_2_, PGF_2α_, PGD_2,_ and 6-keto PGF_1α_ (6kPGF_1α_) were 303 > 259 m/z, 351 > 271 m/z, 353 > 193 m/z, 351 > 271 m/z and 369 > 163 m/z, respectively. Quantification was performed using a standard curve for each compound, and the calculated values were corrected by recovery rates of the internal standard.

### Chemicals and their administration

The COX-2-specific inhibitor celecoxib and the DP agonist BW245C were purchased from Tokyo Chemical Industry and Cayman Chemical, respectively. The EP4 agonist ONO-AE1-329, the EP2 agonist ONO-AE1-259, and the EP4 antagonist ONO-AE3-208 were generous gifts from Ono Pharmaceuticals Co. Ltd.. The specificity of each receptor-targeted drug is summarized in supplemental Table S2. Each receptor agonist was confirmed to have a 100-fold or higher specificity for its target receptor than the other receptors, and the EP4 antagonist was confirmed to have a 10,000-fold or higher specificity for EP4 than the other receptors. For celecoxib administration, celecoxib was dissolved in dimethyl sulfoxide at 1 mg/μl, diluted 20-fold with sesame oil, and administered subcutaneously at 500 mg/kg as reported previously (18). For receptor-specific agonist coadministration, BW245 C, ONO-AE1-329, or ONO-AE1-259 was dissolved in ethanol at 20 mg/ml, diluted 200-fold with sesame oil, and administered subcutaneously at 0.2 mg/kg. For EP4 antagonist administration, ONO-AE3-208 was dissolved in dimethyl sulfoxide at 50 mg/ml, diluted 50-fold with sesame oil, and administered subcutaneously at 10 mg/kg. The specificities of each agonist and antagonist have been confirmed previously, and their doses were set as previously reported (32). Control mice received a sham operation followed by treatment with vehicle. On day 6.5, the spherically swollen areas on the uterine horn were counted as the implantation sites, and then these portions were collected and weighed.

### Generation of EP2 and DP double-knockout (DKO) mice

*Ptgdr* (the gene encoding DP) and *Ptger2* (the gene encoding EP2) are located at a distance of 130 kilobase pairs (kbp) on mouse chromosome 14, making recombination between the two genes unlikely. Therefore, we depleted EP2 from DP-deficient embryonic stem (ES) cells (33) by replacing the coding region of exon 1 of the *Ptger2* gene with the *LacZ* and puromycin-resistant genes and fusing β-galactosidase in-frame after the start of the codon of EP2. Four different DP-deficient ES cell lines with additional EP2 deletions were injected into C57BL/6 blastocysts to obtain chimeric offspring. Finally, two ES lines had DP and EP2 mutations in the same allele, and the other two ES lines had an EP2 mutation in an allele different from that with the DP mutation. DP/EP2 double-deficient mice were backcrossed to C57/BL6 mice, and the N10 generation was used for the experiments. Genotypes of both the *Ptgdr* and *Ptger2* genes in all individual mice were analyzed by genomic PCR using the following three primers in the same tube: (i) 2,716 for *Ptger2*, and DP2-2 for *Ptgdr* as a reverse primer common to wild-type (WT) and knockout (KO) alleles, (ii) 2,701 for *Ptger2*, and DP0-3 for *Ptgdr* as a forward primer specific for the WT allele, and (iii) Puro1 for *Ptger2* and Neo3 for *Ptgdr*; PCR products of 650 bp and 420 bp were detected for WT and KO *Ptger2*, respectively, and products of 730 bp and 430 bp were detected for WT and KO *Ptgdr*, respectively. Primers used for genomic PCR are listed in supplemental Table S3.

### Analysis of female fertility in mutant mice

WT, *Ptgdr*^*−/−*^ (DPKO), *Ptger2*^*−/−*^ (EP2KO), or *Ptgdr*^*−/−*^*Ptger2*^*−/−*^ (DKO) female mice (8- to 12-weeks old) were mated with WT male mice, and the number of pups delivered from each pregnant mother was recorded. As EP2KO mice demonstrate reduced fertility owing to impaired fertilization resulting from defects in the disassembly of cumulus-oocyte matrices (34, 35, 36), DKO mothers were expected to show impaired fertility in a similar manner. Therefore, we evaluated the implantation receptivity of DKO female mice by performing embryo transfer, as follows: pseudopregnancy in WT, DPKO, EP2KO, and DKO recipients was induced by mating them with vasectomized WT males. WT blastocysts (12 for each recipient) were transferred into the uteri of these recipients on day 3.5. On day 13.5, the number of implants were recorded. Alternatively, the recipient mothers were allowed to give birth and the number and weight of the pups born alive were determined. For EP4 antagonist administration, WT fertilized eggs (10 for each recipient) were transferred into the oviducts of WT and DKO recipients on day 0.5. The recipients were injected with the drug on day 4.5 and 5.5, and the number and weight of the implantation site (IS) were measured on day 6.5.

### Statistical analysis

For the statistical analyses of gene expression, PG levels, and the number and weight of implantation sites, a one-way analysis of variance followed by the Student’s or Welch’s *t* test was used to evaluate differences between individual groups. Some statistical analyses were performed after logarithmic transformations of the data, as appropriate. Values were presented as the mean ± standard error of the mean (S.E.M.), and considered to indicate a statistically significant difference at *P* < 0.05.

## Results

### DP mRNA is expressed in the LE from the peri-implantation period to late pregnancy

**Fig. 1:**
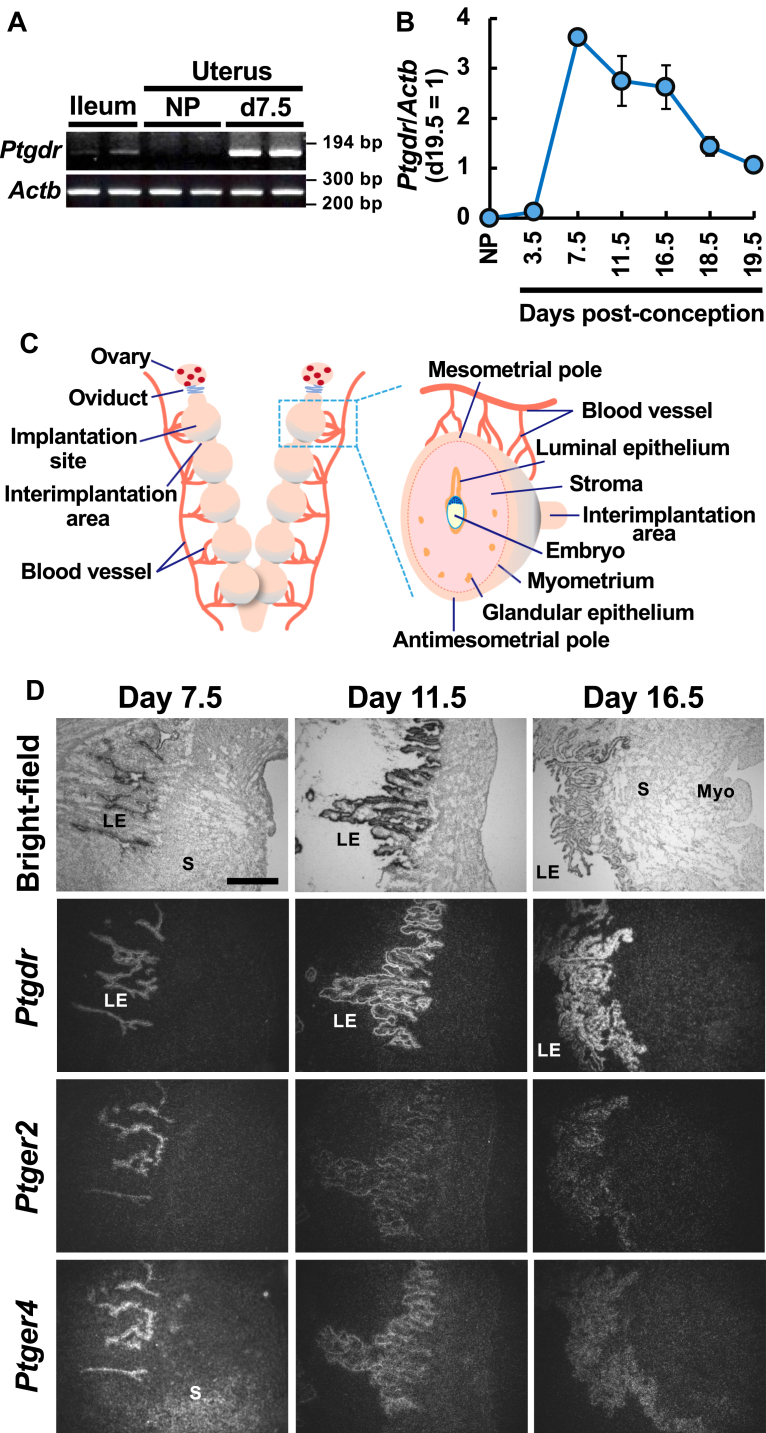
DP receptor is expressed in the mouse uterus during mid to late pregnancy. A: abundant gene expression of DP was detected in uterine tissues of pregnant female mice on day 7.5 post-conception (d7.5), but not in those of nonpregnant female mice by RT-PCR (NP, proestrous). The ileum was used as a positive control. B: quantitative RT-PCR demonstrated that DP gene expression is induced in the uterine tissues of pregnant female mice, peaking on day 7.5 post-conception. Data are shown as the mean ± S.E.M. C: schematic representation of the anatomical structure of the mouse uterus and surrounding tissues (left) and a cross-section of the implantation site (right). D: photomicrographs showing in situ hybridization signals for DP (*Ptgdr*), EP2 (*Ptger2*), and EP4 (*Ptger4*) in interimplantation areas of the uterus on days 7.5, 11.5, and 16.5 post-conception. DP mRNA was expressed in luminal epithelial cells (LE) within this area during mid to late pregnancy. S, stroma; Myo, myometrium. Bar, 500 μm.

To identify the potential sites of PG actions to activate Gs signaling, we investigated the expression of Gs-coupled PG receptors in the uteri of pregnant mice. We found that DP, a Gs-coupled receptor for PGD_2_, is abundantly expressed throughout pregnancy (Fig. 1). RT-PCR demonstrated substantial DP-mRNA expression in the ileum, which is a tissue that has been reported to express DP mRNA (37), whereas no expression of DP mRNA was observed in the uteri of nonpregnant female mice (Fig. 1A). In contrast, DP mRNA expression was found in the uterine tissue of pregnant females on day 7.5 post-conception, when decidual cells are fully matured (7), at a level much higher (approximately 10-fold) than in the ileum, suggesting that DP gene expression is induced by hormonal stimuli associated with pregnancy. We then investigated uterine DP mRNA expression on various gestational days by quantitative RT-PCR in uterine homogenates (Fig. 1B). Whereas no DP expression was detected in the uteri of nonpregnant females, DP expression was induced on day 3.5 post-conception, was substantially increased on day 7.5, and then gradually decreased until day 19.5, the day of expected delivery. As the Gs-coupled PGE receptors EP2 and EP4 are also abundantly expressed in uterine tissue during mid to late pregnancy (24), we then analyzed the spatial expression of DP mRNA, and compared it to those of EP2 and EP4 mRNAs by in situ hybridization on day 7.5 and onwards (Fig. 1C, D). All three PG receptors were detected on LE cells at interimplantation sites from day 7.5 to 16.5. Epithelial expression levels of EP2 and EP4 were highest on day 7.5, and then gradually decreased to day 16.5. In contrast, only DP expression in the epithelial cells was maintained at high levels until day 16.5. These results indicate that the DP receptor is abundantly expressed in LE cells during late pregnancy.

### DP gene expression is induced in LE and the stroma on the mesometrial side in association with implantation

**Fig. 2:**
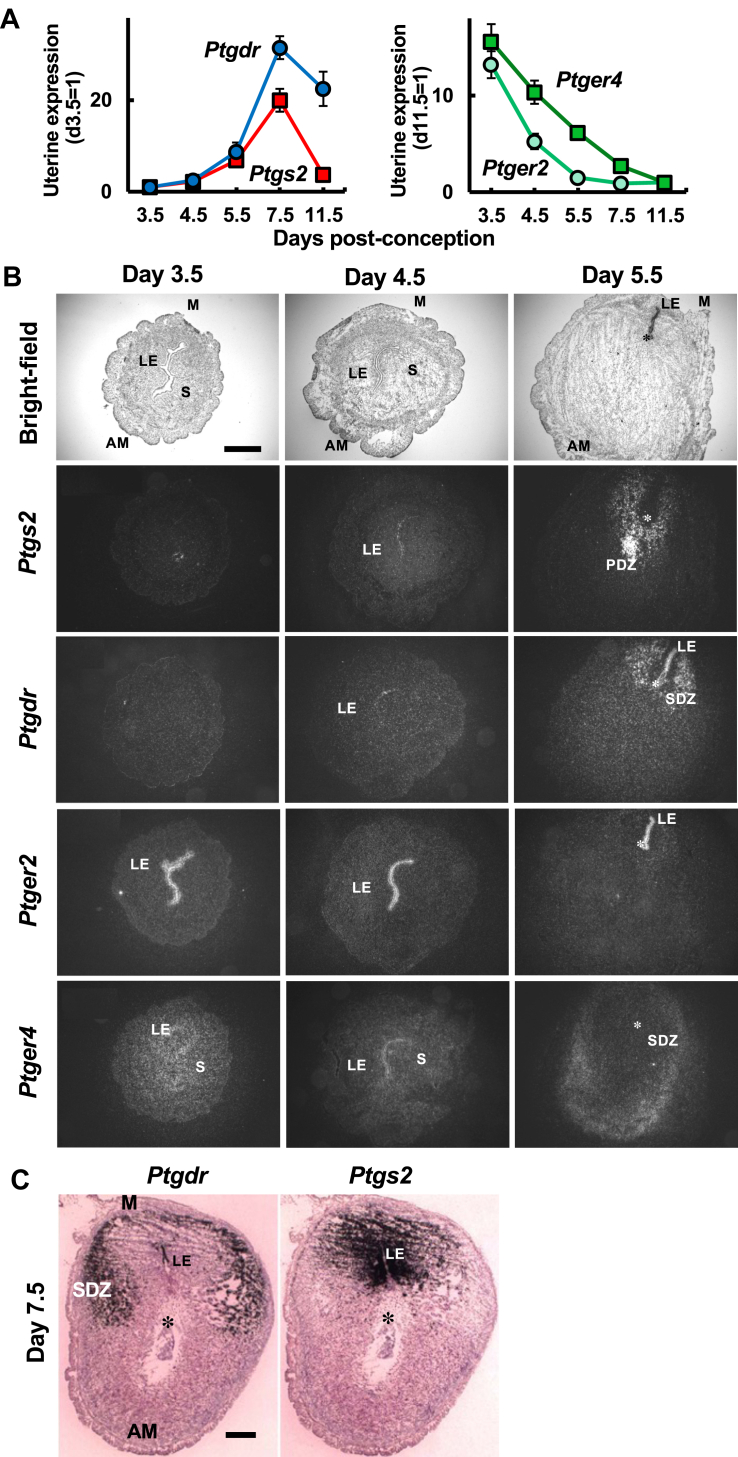
Uterine DP expression is induced upon implantation similarly to COX-2 expression. A: DP (*Ptgdr*) expression was increased in pregnant mouse uteri in a manner similar to COX-2 (*Ptgs2*) expression, from day 4.5 to day 7.5 post-conception, as shown by quantitative RT-PCR. In contrast, uterine expression levels of EP2 (*Ptger2*) and EP4 (*Ptger4*) were rapidly and gradually decreased during this period, respectively. Data are shown as the mean ± S.E.M. B: photomicrographs showing hybridization signals for COX-2 (*Ptgs2*), DP (*Ptgdr*), EP2 (*Ptger2*), and EP4 (*Ptger4*) in the uterus on days 3.5, 4.5, and 5.5 post-conception. C: bright-field photomicrographs showing hybridization signals for DP (*Ptgdr*) and COX-2 (*Ptgs2*) in the uterus on day 7.5 post-conception. DP and COX-2 are expressed in different areas of the mesometrial pole region. Asterisk, embryo; AM, antimesometrial pole; D, decidua; M, mesometrial pole; PDZ, primary decidual zone; S, stroma; SDZ, secondary decidual zone. Bars, 500 μm.

Uterine DP gene expression was first detected on day 3.5 of pregnancy (Fig. 1B). Considering that COX-2 expression is robustly induced in the vicinity of the attached embryo (9), we analyzed the spatiotemporal expressions of the DP and COX-2 genes during the peri-implantation period (Fig. 2A). Uterine COX-2 expression was gradually increased from day 3.5–7.5, and then decreased on day 11.5. Interestingly, uterine DP expression was also increased similarly to COX-2 expression up to day 7.5, and decreased again on day 11.5 (Fig. 2A), suggesting that the COX-2 and DP genes may share similar regulatory mechanisms for their expression. Because the Gs-coupled PGE receptors EP2 and EP4 are known to show unique spatiotemporal expression patterns during the peri-implantation period (24, 25), we analyzed their expressions in the same time frame, and found that the expression levels of EP2 and EP4 were rapidly and gradually decreased during this period, respectively (Fig. 2A). We then compared the expression sites of COX-2, DP, EP2, and EP4 from day 3.5 to 5.5 (Fig. 2B and supplemental Fig. S1). The EP2 receptor was exclusively expressed in LE cells on day 3.5, and such expression was observed until day 5.5. In contrast, EP4 receptor expression was broadly distributed in the endometrial stroma and in LE cells on day 3.5 and 4.5, but shifted to the secondary decidualizing zone (SDZ) within the anti-mesometrial (AM) region on day 5.5. Such EP4 expression in the decidual zone was maintained on day 7.5 (Fig. 1D). COX-2 expression was undetectable on day 3.5, induced in LE cells on day 4.5, and then shifted to the primary decidual zone (PDZ) in the vicinity of embryo-attached sites on day 5.5 (Fig. 2B), as reported previously (15). DP expression was undetectable on day 3.5 but was induced in the LE on day 4.5, and such DP expression was more prominent on day 5.5. Thus, these three Gs-coupled receptors were found to be coexpressed in LE cells, suggesting that such PG receptor signaling may be involved in LE breakdown or reconstruction. On day 5.5, in addition to LE cells, DP expression was induced in stromal cells within the mesometrial (M) region, where COX-2 expression was also observed. Interestingly, such overlapping expression of COX-2 and DP was segregated on day 7.5; COX-2 was observed in the M region with the LE in the center (38), whereas DP was distributed in the SDZ surrounding the site of COX-2 expression (Fig. 2C). Based on these characteristic distribution patterns of COX-2 and DP in the decidual zone, we hypothesized that the paracrine and/or autocrine action of COX-2-derived PGD_2_ may promote the final step of decidualization via the DP-cAMP pathway.

### Both PGD_2_ biosynthesis and specific PGD_2_-binding activity are detected in uterine tissue during the peri-implantation period

**Fig. 3:**
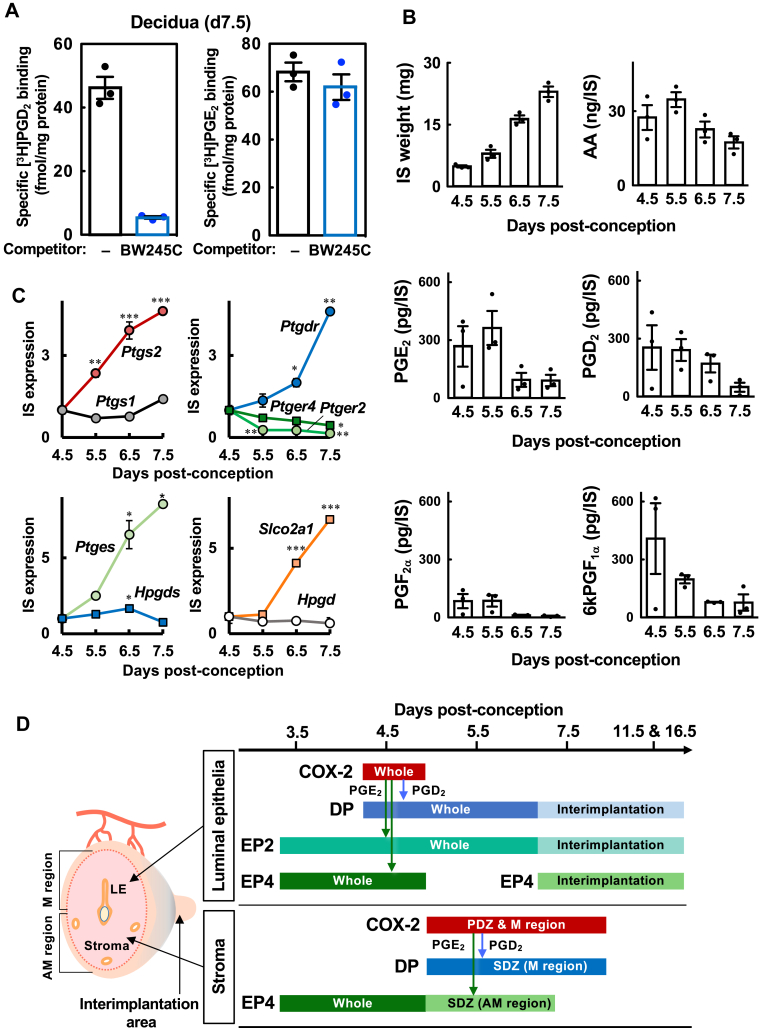
Specific [^3^H]PGD_2_-binding activity and substantial PGD_2_ synthesis are detected in uterine tissues during the decidualization period. A: Specific [^3^H]PGD_2_-or [^3^H]PGE_2_-binding activity in uterine decidual tissues on day 7.5 post-conception. B: Increasing IS weight and amounts of AA, PGE_2_, PGD_2_, PGF_2α_ and 6kPGF_1α_ in IS tissues on day 4.5–7.5 post-conception. C: expression levels of PG-associated genes in IS tissues on day 4.5–7.5 post-conception. Relative expression levels of COX-1 (*Ptgs1*), COX-2 (*Ptgs2*), DP (*Ptgdr*), EP2 (*Ptger2*), EP4 (*Ptger4*), mPGES1 (*Ptges*), H-PGDS (*Hpgds*), PGT (*Slco2a1*), and 15-PGDH (*Hpgd*) are presented with levels on day 4.5 set as 1. Data are shown as the mean ± S.E.M. ∗*P* < 0.05, ∗∗*P* < 0.01, ∗∗∗*P* < 0.005 (versus day 4.5). D: diagram summarizing the results of the expression sites of COX-2 (red), DP (blue), EP2 (blue green), and EP4 (green) in the uterus identified by in situ hybridization. Different colored bars indicate different distribution patterns. Possible COX-2-derived PGD_2_ and PGE_2_ actions are shown as blue and green arrows, respectively.

To further investigate our hypothesis, we first analyzed whether specific PGD_2_ binding activity and PGD_2_ biosynthesis can be detected in the uterine tissue of pregnant mice. We prepared crude membranes isolated from uterine endometrial tissues of pregnant mice on day 7.5, when DP expression was the highest, and performed the PGD_2_ binding assay. As a result, we detected specific PGD_2_-binding activity (46.9 ± 5.8 fmol/mg protein), which was about two-thirds of the specific PGE_2_-binding activity (72.6 ± 5.8 fmol/mg protein) (Fig. 3A). Most importantly, this specific PGD_2_ binding was inhibited in the presence of the specific DP-receptor agonist BW245C, whereas specific PGE_2_ binding was inhibited minimally by this compound. These results demonstrated the existence of functional DP receptors in the cell membranes of uterine endometrial tissues undergoing terminal decidualization. We then measured PG contents in individual IS tissues during the peri-implantation period. During this period, the IS tissues grew rapidly, but AA content did not change significantly (Fig. 3B). A substantial amount of PGE_2_ was produced in the IS tissues on days 4.5 and 5.5, but PGE_2_ synthesis was lowered on days 6.5 and 7.5 (Fig. 3B). Intriguingly, a large amount of PGD_2_, comparable to PGE_2_, was also detected in the IS tissues on days 4.5, 5.5 and 6.5, but such a PGD_2_ synthesis was decreased on day 7.5. In contrast, PGF_2α_ levels were less than half those of PGE_2_ and PGD_2_ in the IS tissues. A stable PGI_2_ metabolite, 6kPGF_1α_, was also abundantly detected on day 4.5 but decreased sharply on day 5.5 and further on days 6.5 and 7.5. Thus, PGD_2_ is biosynthesized in amounts comparable to PGE_2_ in IS tissues. This indicates that in addition to the PGE_2_-EP axis, the PGD_2_-DP receptor signaling pathway also works during the peri-implantation period. To further gain insight into PG synthesis and metabolism during the peri-implantation period, we investigated the expression of PG-associated genes in IS tissues in the same time frame as PG quantification (Fig. 3C). COX-2 (*Ptgs2*) expression was increased throughout the period, as was DP expression, whereas COX-1 (*Ptgs1*) and EP4 (*Ptger4*) expression remained largely unchanged, and EP2 (*Ptger2*) expression was rapidly decreased. Similar to COX-2 expression, microsomal PGE synthase-1 (mPGES1, *Ptges*) expression was also increased, and hematopoietic PGD synthase (H-PGDS, *Hpgds*) expression was also increased to 2-fold on day 6.5. Interestingly, the expression of PG transporter (PGT, *Slco2a1*) was abruptly increased from day 6.5 to 7.5, whereas expression of the PG-inactivating enzyme 15-hydroxyprostaglandin dehydrogenase (15-PGDH, *Hpgd*) remained largely unchanged. These results suggest that implantation-induced COX-2 and PG synthases cooperatively synthesize PGE_2_ and PGD_2_ to maintain them at high levels within IS tissues up to day 6.5, after which cellular uptake by PGT and subsequent metabolism by 15-PGDH appear to reduce total PG levels within the IS tissue. The expression sites of COX-2, DP, EP2, and EP4 in the uterine tissues detected by in situ hybridization are schematically summarized in Fig. 3D. As shown, DP, EP2 and EP4 are coexpressed in the LE at day 4.5, when decidualization is initiated and both PGD_2_ and PGE_2_ are abundantly synthesized. In the stroma, in addition to extensive expression of the EP4 receptor, the DP receptor is induced in a temporally parallel manner with COX-2 expression, but is expressed in cells distinct from COX-2 sites, leading us to hypothesize that PGD_2_ synthesized by COX-2 promotes stromal differentiation by acting on DP in the surrounding cells in a paracrine manner.

### Decidualization is suppressed by pharmacological COX-2 inhibition but rescued by DP receptor activation

**Fig. 4:**
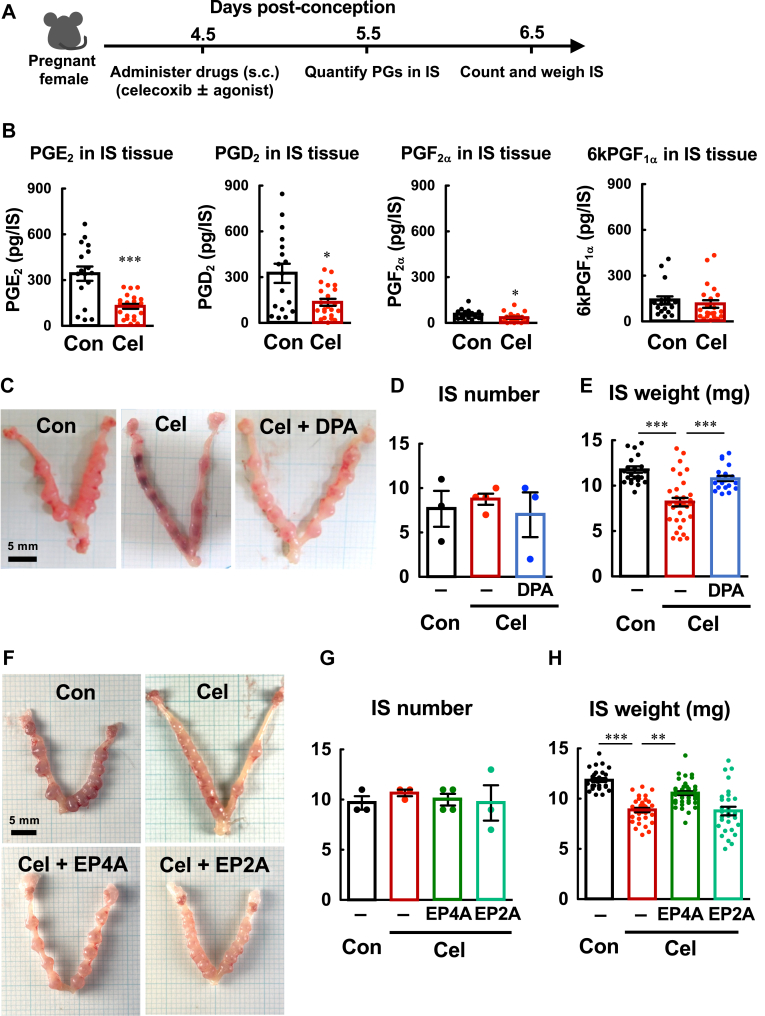
Administration of a DP or an EP4 agonist, but not an EP2 agonist rescues celecoxib-elicited growth failure in IS tissues. A: schematic representation of the experimental design. Vehicle (Con) or celecoxib (Cel, 500 mg/kg) in the presence or absence of BW245 C (DPA, 0.2 mg/kg), ONO-AE1-259 (EP2A, 0.2 mg/kg) ONO-AE1-329 (EP4A, 0.2 mg/kg) was administered subcutaneously (s.c.) on day 4.5 post-conception, PG contents in IS tissues were measured on day 5.5 post-conception, and the uteri were analyzed on day 6.5 post-conception. B: suppression of PG synthesis in IS tissues by celecoxib. *C*: the representative appearance of the uterine tissues of various mice. D and E: effects of celecoxib in the presence or absence of the DP agonist on the number (D) and weight (E) of IS tissues. F–H: rescuing ability of an EP4 or EP2 agonist on celecoxib-induced growth failure in IS tissues. Appearance of the uterine tissues (F), and the number (G) and weight (H) of IS tissues are shown. Data are shown as the mean ± S.E.M. ∗*P* < 0.05, ∗∗*P* < 0.01, ∗∗∗*P* < 0.005.

As COX-2-deficient mice show defects in implantation and decidualization, COX-2 is important for the PG synthesis that is required to accomplish these processes. Therefore, we first blocked decidualization by administration of the COX-2-specific inhibitor celecoxib and then analyzed whether pharmacological DP or EP activation can rescue it. Pregnant female mice were injected with celecoxib on day 4.5 just after embryo attachment, PG contents in IS tissues were analyzed on day 5.5, and the number and weight of the IS tissues were evaluated on day 6.5 (Fig. 4A). Indeed, celecoxib efficiently attenuated both PGE_2_ and PGD_2_ levels, but not 6kPGF_1α_ level within IS tissues on day 5.5 (Fig. 4B). Although celecoxib did not affect the number of IS tissues, this drug significantly reduced the weight of the IS tissues (Fig. 4C–E), suggesting that the decidualization of stromal cells is impaired by COX-2 inhibition. Most importantly, BW245 C completely reversed the suppressive effects of celecoxib (Fig. 4C–E). When we analyzed the effects of various EP-specific agonists, we found that an EP4 agonist, but not an EP2 agonist, also restored IS weight in the celecoxib-treated mice (Fig. 4F–H). These results indicate that at least the PGD_2_-DP and PGE_2_-EP4 pathways contribute to decidualization, presumably by stimulating cAMP signaling and stromal cell differentiation.

### DP/EP2 double deficiency does not affect implantation and decidualization

**Fig. 5:**
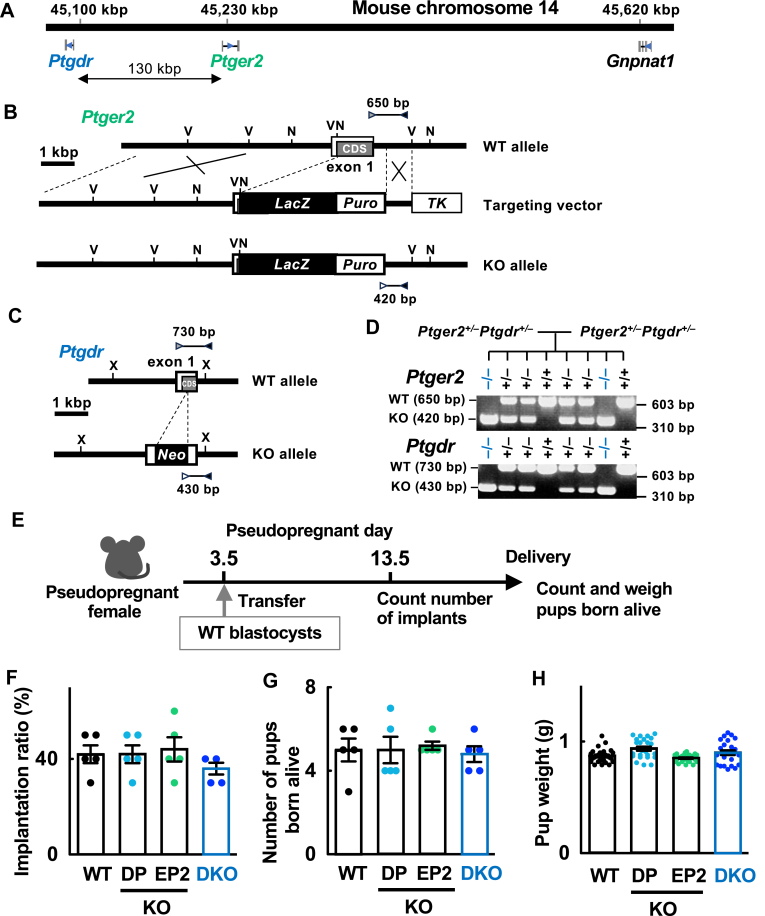
DP-, EP2-, and EP2/DP-deficiency do not affect the implantation ability of female mice. A: *Ptgdr* and *Ptger2* genes on mouse chromosome 14 are located at a distance of 130 kbp, making recombination between them unlikely. *Gnpnat1*, gene encoding glucosamine 6-phosphate N-acetyltransferase. B: Diagram showing the strategy for *Ptger2* gene targeting in the *Ptgdr*^*+/−*^ ES cell line, which is resistant to neomycin. ES cells with homologous recombination in the *Ptger2* gene were designed to be selected for puromycin and ganciclovir resistance. Construction of the targeting vector, organization of the *Ptger2* gene, and structure of the targeted genome are shown with primer positions used for genomic PCR and the amplified fragment of each allele. Restriction sites are indicated as follows: N, *Nco*I; V, *Eco*RV. C: organization of the mouse *Ptgdr* gene and the structure of the mutated genome are shown together with the primer positions used for genomic PCR, and the amplified fragment of each allele. *Neo*, neomycin-resistant gene; X, *Xba*I restriction site. D: Representative results of genomic PCR analysis of litters born from double heterozygous crosses. E: Schematic representation of embryo transfer and evaluation of maternal implantation receptivity. F–H: implantation of WT blastocysts transferred to pseudopregnant WT, DPKO, EP2KO, or DKO mice (n = 5). WT blastocysts (12 for each) were transferred into the uteri of the indicated mice on day 3.5 of pseudopregnancy. Recipients were sacrificed on day 13.5 to check the implants (F). Alternatively, the recipients were allowed to give birth to determine the number (G) and weight of pups born alive (H). Data are shown as the mean ± S.E.M. bp, base pairs; CDS, coding sequence; kbp, kilobase pairs; *LacZ*, β-galactosidase gene; *Puro*, puromycin-resistant gene; *TK*, thymidine kinase gene.

As activation of DP or EP4 on its own was found to contribute to COX-2-dependent decidualization, we considered that these Gs-coupled PG receptors compensate for each other in the stimulation of implantation-associated processes. To assess our hypothesis, it would be ideal to analyze DP/EP2/EP4 triple-deficient mice, but it is difficult to analyze mice with a genetic background of EP4 deficiency, owing to neonatal lethality (39, 40). Therefore, we first investigated whether the double deficiency of DP and EP2 affects implantation and decidualization. As the DP and EP2 genes are positioned close to each other on mouse chromosome 14 at a distance of 130 kbp (Fig. 5A), and recombination between them was considered to be unlikely, we introduced an additional EP2 deletion into the heterozygous DPKO ES cell line (33). As DP-mutated cells are resistant to neomycin, we constructed a targeting vector so that the coding region of the first exon of the EP2 receptor gene could be replaced with the *LacZ* and puromycin-resistant genes, and finally established DP/EP2 DKO mice (Fig. 5B–D). Heterozygous crosses resulted in DKO mice that were born at the expected Mendelian ratio (Fig. 5D), suggesting that DKO mice are not embryonic lethal. We first analyzed the litter sizes of DPKO, EP2KO, and DKO female mice by mating them with WT male mice (supplemental Table S4). DPKO mice had litter sizes that were comparable to those of WT mice. As previously reported, EP2KO mice have impaired fertilization owing to the abortive disassembly of cumulus matrices (34, 35, 36), causing low litter sizes in EP2KO dams. The litter sizes of DKO mice were similar to those of EP2KO mice, suggesting that DP ablation did not reduce fertility. We also tested implantation rates by transferring WT blastocysts into female mice, to eliminate the effects of defective ovulation/fertilization by EP2 deficiency (Fig. 5E). All of the mutant females showed implantation rates similar to those of WT mice, and gave birth to viable pups with similar weights (Fig. 5F–H). As DP/EP2 deficiency did not affect implantation and subsequent fetal growth, we concluded that this mutation does not intrinsically affect decidualization either. During the peri-implantation period, in addition to DP and EP2, the EP4 receptor is also expressed in LE cells. On the other hand, in stromal cells, in addition to DP, EP4 is also expressed more extensively during this period (Fig. 3D). Thus, even in the absence of DP and EP2 receptors, EP4 receptor signaling alone in the epithelia or stroma may be sufficient to accomplish epithelial breakdown and decidualization, respectively.

### Pharmacological ablation of EP4 signaling in DP/EP2 DKO mice results in decidualization failure

**Fig. 6:**
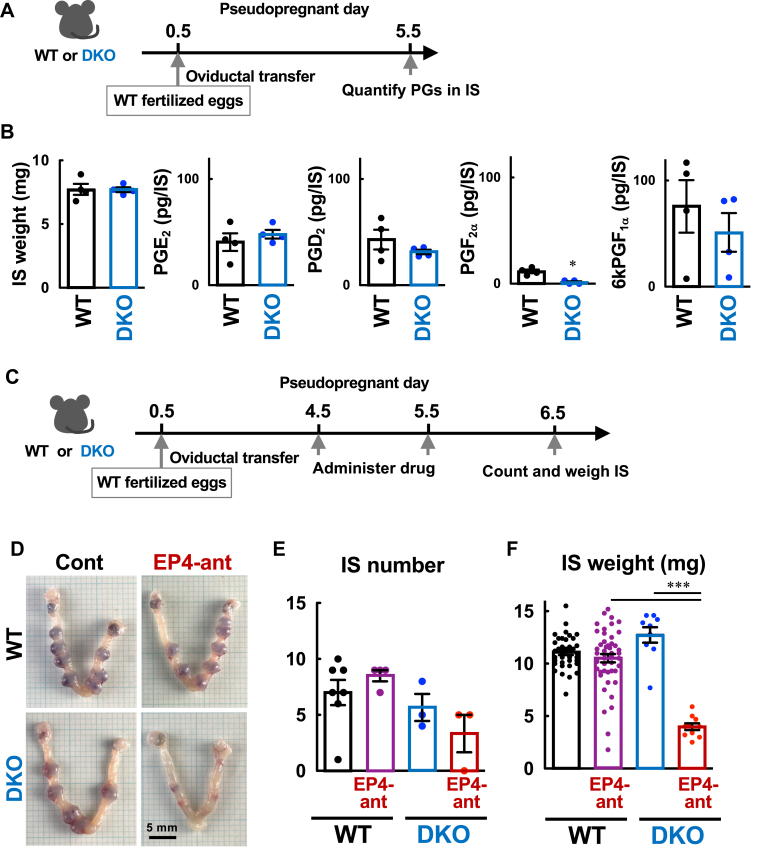
Ablation of EP4 signaling in DP/EP2 DKO mice results in severe disruption of IS growth. A: schematic representation of the experimental design. WT or DP/EP2 DKO female mice on day 0.5 of pseudopregnancy were subjected to oviductal transfer of WT fertilized eggs (10 for each), and PG contents in IS tissues were measured on day 5.5. B: weight and amounts of PGE_2_, PGD_2_, PGF_2α_, and 6kPGF_1α_ in IS tissues isolated from WT and DKO mice on day 5.5. C: WT or DKO female mice that received an oviductal transfer of WT fertilized eggs, as shown in A, were administered an EP4 antagonist (EP4-ant, 10 mg/kg, s.c.) on days 4.5 and 5.5, and uteri were analyzed on day 6.5. D–F: Blockage of EP2/EP4/DP receptor signaling led to severe disruption of IS growth. Appearance of the uterine tissues (D), and the number (E) and weight (F) of IS tissues are shown. Data are shown as the mean ± S.E.M. ∗*P* < 0.05, ∗∗∗*P* < 0.005.

To test our hypothesis described above, we investigated the effects of an EP4 antagonist on decidualization in DKO female mice. To maintain the intrauterine environment as close as possible to that of spontaneous ovulation, we performed the oviductal transfer of WT fertilized eggs and checked whether PGs in IS tissue were synthesized in DKO mice to the same extent as in WT mice (Fig. 6A, B). The results showed that PGE_2_, PGD_2_, and 6kPGF_1α_ were abundantly detected in IS tissues of DKO mice on day 5.5, with levels similar to WT mice. Therefore, the loss of EP2 and DP receptor signaling is unlikely to significantly affect PG synthesis during this period. We then administered WT and DKO recipients with an EP4 antagonist on days 4.5 and 5.5, to perturb endogenous PGE_2_-EP4 signaling during decidualization (Fig. 6C–F). Our results demonstrated that EP4 blockage did not affect the IS number of either WT or DKO recipients, nor did it affect the IS weight of WT recipients, but it severely reduced the IS weight of DKO recipients; that is, the lack of EP4 signaling in DKO recipients elicited severe implantation failure, and such suppressive effects appeared more potent than those of the COX-2 inhibitor in WT females. Based on these findings, we conclude that PGD_2_-DP receptor signaling physiologically contributes to decidualization, compensating for the PGE_2_-EP4 pathway.

## Discussion

### PGD_2_-DP pathway was the missing piece of the activity of PG receptors in uterine tissues

**Fig. 7:**
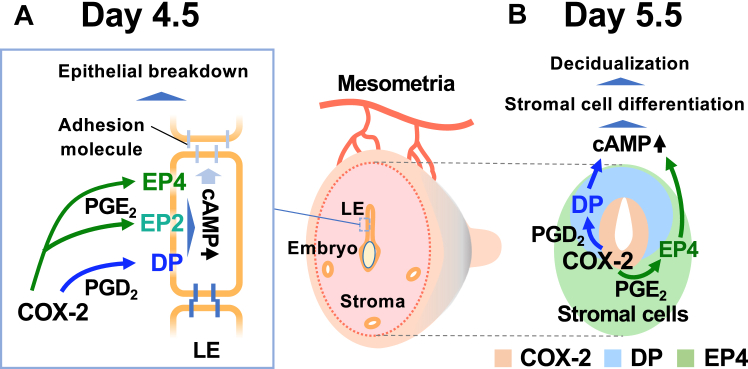
Schematic representation of the roles of the PGD_2_-DP and PGE_2_-EP4/EP2 receptor axes in implantation and decidualization. A: three Gs-coupled PG receptors, DP, EP2, and EP4, are expressed in the LE on day 4.5 (the time of implantation). COX-2-derived PGD_2_ and PGE_2_ are assumed to promote epithelial breakdown by affecting adhesion molecules in a coordinated manner, via DP, EP2, and EP4 receptors in the LE. B: on day 5.5, PGs synthesized by COX-2 promote decidualization by stimulating cAMP-dependent stromal cell differentiation in the M and AM pole regions via the PGD_2_-DP and PGE_2_-EP4 axes, respectively.

Because COX-2 deficiency impairs both implantation and decidualization, PGs have been suggested to play an essential role in these processes. However, as mice deficient in each of the eight PG receptors do not show abnormalities in implantation-associated events (26), the type of PG receptors involved in these processes has long been a mystery. In this study, we found that functional DP receptors are expressed concomitantly with the synthesis of a substantial amount of PGD_2_ in uterine tissue during the peri-implantation period; ie, the PGD_2_-DP receptor axis was the missing piece of the “PG receptor puzzle” of implantation. We also found that three Gs-coupled PG receptors, DP, EP2, and EP4, are expressed in LE cells, and thereafter, DP and EP4 receptors are expressed in stromal cells within the M and AM regions, respectively, during the peri-implantation period (Fig. 3D). Therefore, we focused on DP, EP2, and EP4 receptors, and evaluated their contribution to decidualization by pharmacological and genetic approaches. Although DP/EP2 double-deficiency did not affect the decidualization, the presence of an EP4 antagonist in the DP/EP2-null condition led to severe defects in decidualization (Fig. 6). Moreover, impaired decidualization induced by COX-2 blockage was restored by the administration of a DP or EP4 agonist, but not an EP2 agonist. Such results with a clear contrast in agonist efficacy indicate that both the DP and EP4 receptor pathways have the ability to promote decidualization. Celecoxib reduced both PGD_2_ and PGE_2_ syntheses to approximately 40%, but did not completely eliminate the decidualizing cAMP signals of the DP and EP4 pathways, suggesting that the activation of either receptor could have compensated for the deficient cAMP signals in stromal cells. Based on these results, we concluded that at least the PGD_2_-DP and PGE_2_-EP4 pathways are cooperatively involved in decidualization (Fig. 7B).

### PGD_2_-DP and PGE_2_-EP4 pathways are cooperatively involved in decidualization

In this study, we demonstrate that the PGD_2_-DP and PGE_2_-EP4 pathways are involved in decidualization. Indeed, a previous report on the analysis of human endometrial stromal cells in vitro showed that progesterone-induced decidualization is dependent on PG-induced cAMP signaling (19). Moreover, COX-2-derived PGE_2_ has been shown to elicit decidual reactions by upregulating stromal cAMP levels via the EP4 receptor (41). Aikawa *et al.* further demonstrated that COX-2-derived PGs play a pivotal role in the process of stromal cell differentiation in LPAR3 agonist-induced decidualization (18). Then, how do the DP and EP4 pathways individually participate in decidualization? As mentioned above, on day 5.5 post-conception, DP and EP4 are expressed in stromal cells within the M and AM regions, respectively (Fig. 3D). Such a difference between the localization of DP and EP4 may reflect different sites of PGD_2_ and PGE_2_ synthesis; i.e., PGD_2_ synthesis within the IS remains high up to day 6.5 when the M pole stroma proliferates most efficiently, whereas PGE_2_ synthesis is already reduced by day 6.5, suggesting that PGD_2_ and PGE_2_ are likely synthesized within the M and AM regions, respectively. As reviewed by Das (4), stromal cells within the AM region are heterologous in nature, with some being proliferative and others differentiated, whereas stromal cells within the M region are uniformly proliferative in terms of their expression of cyclin D3 and cyclin-dependent kinase CDK4. Therefore, in these proliferative stromal cells within the M region, the PGD_2_-DP pathway exclusively activates cAMP signaling, and thereby locally promotes collective differentiation. In contrast, to differentiate the heterologous stromal cells within the AM region, PGE_2_ may need to activate other EPs (such as EP1 and EP3) in addition to Gs-coupled EP4, for example to regulate uterine tension. Indeed, the rescue effect of an EP4 agonist on decidualization failure by COX-2 inhibition was less potent than that of a DP agonist (Fig. 4), which may be owing to the lack of EP activation other than EP4. Thus, PGE_2_ appears to systematically promote decidualization through multiple receptors.

### PG receptor signaling in LE breakdown

Once the embryo attaches to LE cells, tight junctions between LE cells in the vicinity of the embryo are weakened, promoting trophoblast invasion into the decidua (42). Aikawa *et al.* showed that LE breakdown is also sensitive to a COX-2 inhibitor, suggesting that PG signaling also participates in this process (18). This study revealed that DP, EP2, and EP4 receptors are expressed in LE cells on day 4.5 (Fig. 3D), suggesting that three PG receptors may contribute to triggering LE breakdown. The mechanism regulating epithelial junctions by the PG receptor-cAMP pathway was characterized in an experimental system of competitive exclusion of yes-associated protein- (YAP-) expressing MDCK cells (43). COX-2 expression is induced in the epithelial cells to be eliminated, and COX-2-derived PGE_2_ acts on the EP2 receptor expressed in the PG-producing cells themselves and in adjacent cells, to elicit the internalization of E-cadherin via the cAMP/protein kinase A (PKA) pathway; this causes COX-2-expressing cells to lose their epithelial junctions and be eliminated. Indeed, YAP has been reported to be present in the LE (44), and hence a similar mechanism may facilitate LE breakdown by the coordinated actions of three Gs-coupled PG receptors (Fig. 7A). As the conditions under which COX-2 inhibitors were administered in this study did not affect the number of IS, it would be necessary to optimize the conditions that inhibit implantation or to analyze the effects of uterus-specific receptor deficiency, to clarify the contribution of these PG receptors in LE breakdown.

### Synthesis and metabolic clearance of PGs within IS tissue

The association between the dynamics of PGs and the expression patterns of PG synthase genes within IS tissue indicate that PGD_2_ and PGE_2_ are synthesized downstream of COX-2 by the actions of H-PGDS and mPGES1, respectively, because both *Hpgds* and *Ptges* expression levels were increased on day 6.5 (Fig. 3B, C). Interestingly, the expression of the PGT gene (*Slco2a1*) was also induced on day 6.5, and further increased on day 7.5. It has been demonstrated that PGT is involved in the metabolic clearance of PGs, including PGE_2_ and PGD_2_, which are taken up into the cells by PGT, and then inactivated through oxidation of their 15-hydroxyl group by 15-PGDH that is expressed in the same cells (45, 46). Indeed, it was reported that *Slco2a1* expression and PGE_2_ uptake activity are highly up-regulated upon cAMP-induced decidualization in endometrial stromal cells (47). Therefore, PGT may contribute to the metabolic clearance of PGE_2_ and PGD_2_ by decidualized cells within the IS tissue. On the other hand, the PGI_2_ metabolite 6kPGF_1α_ was abundantly detected in IS tissue on day 4.5, as previously reported (48), suggesting that PGI_2_ may also be involved in the implantation process, such as in LE breakdown. However, under the present experimental conditions, COX-2 inhibition reduced the levels of both PGE_2_ and PGD_2_, but not 6kPGF_1α_ in IS tissues on day 5.5 (Fig. 4B), suggesting that PGI_2_ is unlikely to be involved in decidualization.

### Transcriptional regulation of Ptgdr and Ptgs2 genes

Our present study demonstrated that the DP gene undergoes a very similar time course of expression induction as the COX-2 gene, although the cell types which they are expressed are different. Therefore, it is possible that the DP and COX-2 genes share common mechanisms in their transcriptional regulation in uterine cells. Stromal COX-2 expression was shown to be induced in a p38 mitogen-activated protein (MAP) kinase-dependent manner during decidualization, suggesting that the transcription factors acting downstream of p38 MAP kinase, including cAMP-responsive element binding protein (CREB) and CCAAT/enhancer binding protein (C/EBP), are involved in the induction of COX-2 (49). When we analyzed the transcription factor binding profiles of the upstream sequences of the COX-2 and DP genes, we found that the promoter and enhancer regions of both genes include potential binding sites of CREB and C/EBPs (supplemental Fig. S2). Moreover, uterine chromatin immunoprecipitation sequencing analyses demonstrated that the progesterone receptor and estrogen receptor also bind to these regions (GSE86314 and GSE137196). Therefore, it is plausible that COX-2 and DP genes share common mechanisms in their transcriptional regulation in uterine cells during the peri-implantation phase.

### Roles of Gs-coupled PG receptors in the LE of interimplantation sites

Interestingly, the three Gs-coupled PG receptors continue to be expressed in the LE of interimplantation sites from day 7.5 post-conception to late pregnancy. The precise role of PGs in the LE during late pregnancy has received minimal attention because of their major role in luteal regression (PGF_2α_) and myometrial contraction (PGE_2_), both of which are prerequisites for successful parturition (50, 51). However, PGs may be involved in the stimulation of basal mucus secretion from the uterine epithelia in a cAMP-dependent manner as suggested previously (52). A recent study based on the systematic analysis of PKA signaling in various epithelial cells indicated that the cAMP-PKA signaling pathway regulates the sensitivity against extracellular stimuli and transport activity of epithelial cells (53). Therefore, these PG receptors may modulate mucus secretion as a defense system in the uterus during pregnancy.

In summary, we found that the functional DP receptor is expressed concomitantly with the synthesis of a substantial amount of PGD_2_ in uterine tissue during the peri-implantation period, that the Gs-coupled PG receptors, DP, EP2, and EP4 are expressed in LE cells on day 4.5, and that DP and EP4 are expressed in stromal cells within the M and AM regions, respectively, on day 5.5. DP/EP2 double deficiency did not affect decidualization, but EP4 signaling blockage in the DP/EP2-null condition elicited a severe failure in decidualization. The impaired decidualization by COX-2 blockage was restored by DP or EP4 activation but not by EP2 activation. Taken together, we conclude that the PGD_2_-DP and PGE_2_-EP4 pathways, and possibly other pathways, are cooperatively involved in decidualization.

### Limitations of the study

In this study, we demonstrated the elimination of all three Gs-coupled PG receptors. namely, DP, EP2, and EP4 receptors result in severe decidualization failure, and impaired decidualization caused by COX-2 blockage is rescued by the activation of DP or EP4, but not by the activation of EP2. However, we did not directly demonstrate in the present study that the depletion of both DP and EP4 axes on their own leads to a severe decidualization defect. Moreover, we did not investigate whether DP, EP2, and EP4 are cooperatively involved in LE breakdown. Further experiments will be needed in the future to clarify the exact contribution of these Gs-coupled PG receptors to each of the implantation-associated processes.

## Data availability

All data associated with this study are presented within the manuscript.

## Supplemental data

This article contains supplemental data.

## Conflicts of interest

The authors declare that they have no conflicts of interest regarding the contents of this article.
