# Supplementary material for: Uterine prostaglandin DP receptor-induced upon implantation contributes to decidualization together with EP4 receptor
Source: J Lipid Res. 2024 Aug 31;65(10):100636. doi: 10.1016/j.jlr.2024.100636 (PMC11465058; doi:10.1016/j.jlr.2024.100636)
Supplement: Supplemental data [file mmc1.pdf]

Uterine prostaglandin DP receptor induced upon implantation contributes to decidualization together with EP4 receptor

Risa Sakamoto, Takuji Fujiwara, Yuko Kawano, Shizu Aikawa, Tomoaki Inazumi, On Nakayama, Yukiko Kawasaki-Shirata, Miho Hashimoto-Iwasaki, Toshiko Sugimoto, Soken Tsuchiya, Satohiro Nakao, Toru Takeo, Yasushi Hirota, and Yukihiko Sugimoto

Supplementary Tables and Figures

Table S1. Primer sequences used for real time RT-PCR

Product	Gene	Forward (5′ to 3′)	Reverse (5′ to 3′)
COX-2	*Ptgs2*	TTCTACGGAGAGAGTTCATCCCT	TTGTCTGTCCAGAGTTTCACCA
COX-1	*Ptgs1*	CCTTGGCCACATTTATGGAG	AAGCAACCCAAACACCTCCT
DP	*Ptgdr*	CCCAGTCAGGCTCAGACTAC	CTTCTGAGTCTCCTTCAGCTT
EP2	*Ptger2*	CCATTATGACCATCACCTTCGC	GCCTAAGTATGGCAAAGACCCAA
EP4	*Ptger4*	TGCTCCATTCCGCTCGT	GCACAGTCTTCCGAAGAAGG
mPGES1	*Ptges*	GGCCTCCAGTATTACAGGAGTG	CGAGGAAATGTATCCAGGCGA
H-PGDS	*Hpgds*	GGAAGAGCCGAAATTATTCGCTAC	TGGTCAAATATCTTGCTATTGCGAG
PGT	*Slco2a1*	TCCTCAGGCTTCTTGATCGT	CAGCATGAAGAGTGGGTTCA
15-PGDH	*Hpgd*	AACGGAGGTGAAGGCG	CCTTTCGATGCCGTGATCT
β-actin	*Actb*	CCTGTATGCCTCTGGTCGTA	CCATCTCCTGCTCGAAGTCT

Table S2. Specificity of each receptor-specific drug used in this study

Drug	Description	Binding (Ki, μM)		
		DP	EP2	EP4
BW245C	DP agonist	0.00126	0.22	0.17
ONO-AE1-329	EP4 agonist	>10	2.1	0.0097
ONO-AE1-259	EP2 agonist	0.41	0.003	6.0
ONO-AE3-208	EP4 antagonist	>10	>10	0.0013

The concentration of each drug that inhibits binding of the endogenous ligand to each receptor by 50% is expressed as a Ki value (μM). The agonist specific for each receptor has a 100-fold or higher specificity for its target receptor than the other receptors; e.g., the EP4 antagonist demonstrates a more than 10,000-fold higher specificity for EP4 than the other receptors.

Table S3. Primer sequences used for genomic PCR

Gene	Primer	Sequence (5′ to 3′)	Notes
Ptger2	2701	CTGGCCATTATGACCATCACCTTCGCCA	Forward primer specific for WT allele
	Puro1	TAATTCCATCAGAAGCTGGTCGACCTCG	Forward primer specific for KO allele
	2716	CTGAGCAACACCCATGTTTCTATCCTGG	Reverse primer common to both alleles
Ptgdr	DP0-3	AGCCTAGATAGGAGGGCTGACTGTGAAT	Forward primer specific for WT allele
	Neo3	TGCCGAATATCATGGTGGAAAATGGCCG	Forward primer specific for KO allele
	DP2-2	TCCTTGTGGATCATCTGGATGAAACACC	Reverse primer common to both alleles

Table S4. Litter sizes of WT, DPKO, EP2KO, and DKO mothers

Genotype	WT	DPKO	EP2KO	DKO
Litter size	7.0 ± 0.82	7.1 ± 0.91	1.4 ± 0.50*	1.3 ± 0.47*

Litter sizes are presented as the mean ± S.E.M. ($n = 6$). *$P < 0.01$ *versus* WT

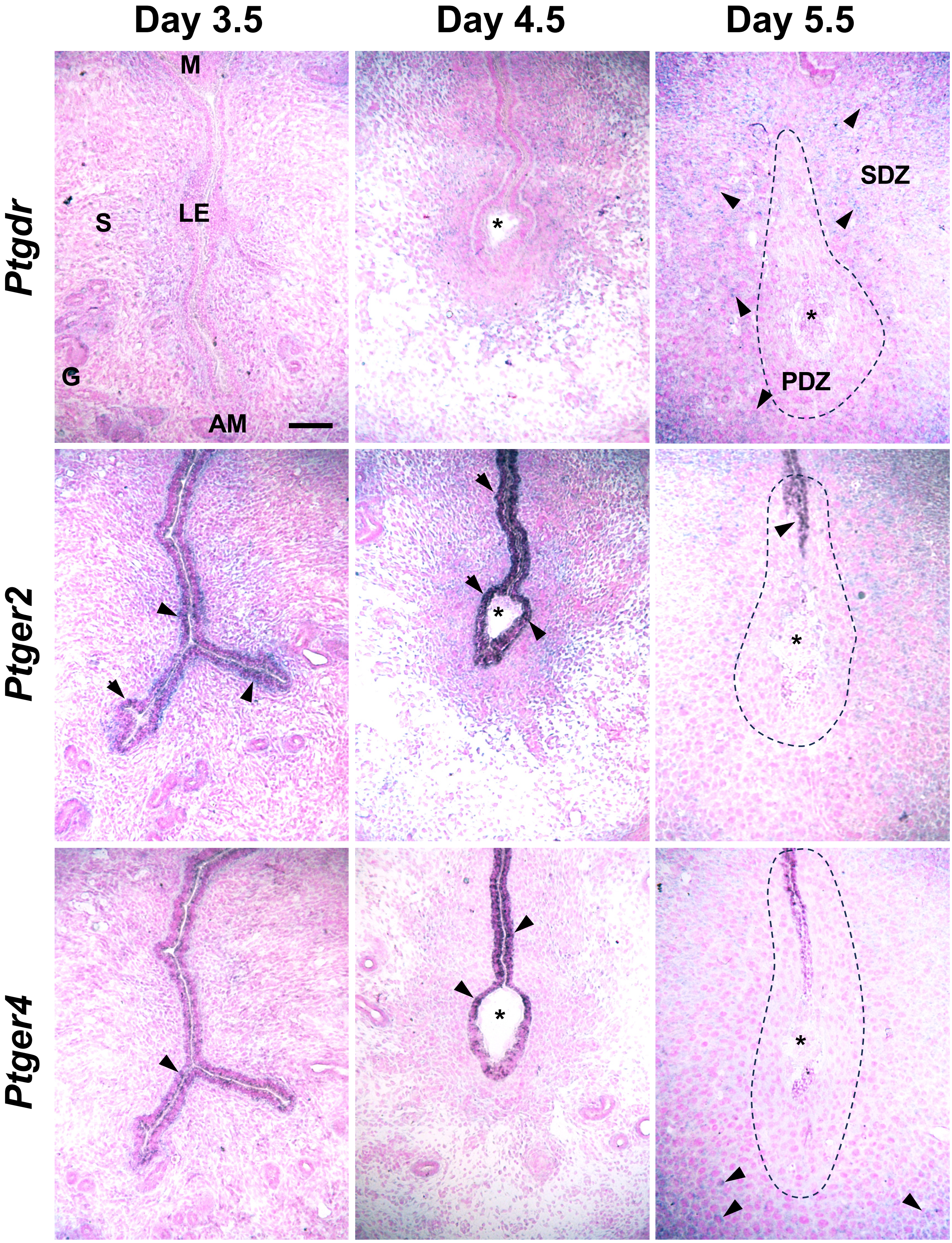

Figure S1. Bright-field photomicrographs showing hybridization signals for *Ptgdr*, *Ptger2*, and *Ptger4*, in the uterus on days 3.5, 4.5, and 5.5 post-conception. Arrowheads indicate hybridization-positive cells labeled with blue hybridization signals. Asterisks represent embryos. In the photomicrographs for day 5.5, the areas enclosed by the dotted lines represent the PDZ, and the areas outside represent the SDZ. AM, antimesometrial pole; D, decidua; G, glandular epithelium; LE, luminal epithelium; M, mesometrial pole; PDZ, primary decidual zone; S, stroma; SDZ, secondary decidual zone. Bar, 100 μm

Sakamoto et al.
Figure S1

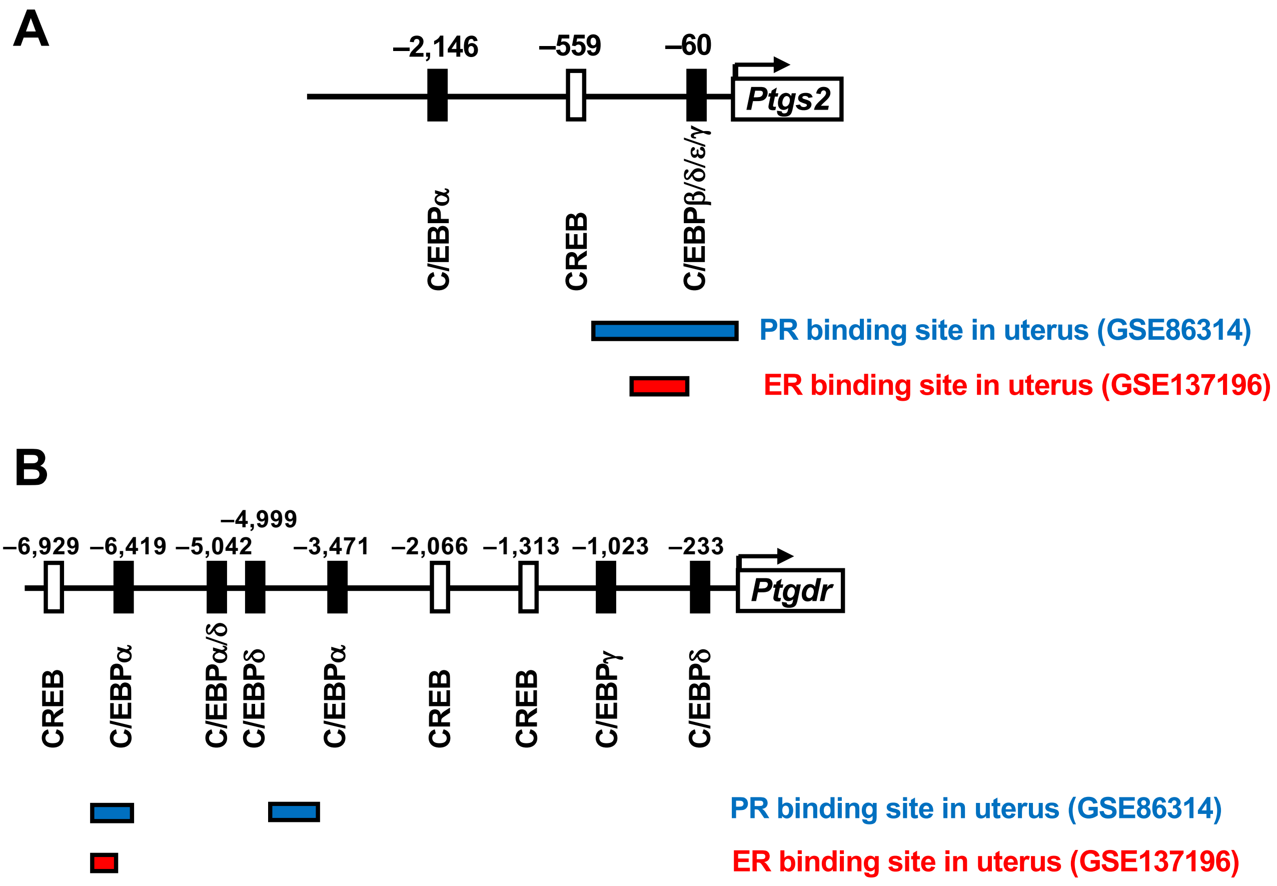

Figure S2. Distribution of transcription factor-binding sites in upstream regions of the COX-2 gene (A) and the DP gene (B). Potential binding motifs of CREB (open boxes) and C/EBPs (closed boxes) were searched using the JASPAR CORE database. Blue and red bars indicate PR-binding and ER-binding sites, respectively, in cells within the uterus identified by chromatin immunoprecipitation-sequencing analysis. C/EBP, CCAAT/enhancer binding protein; CREB, cAMP-responsive element binding protein; ER, estrogen receptor; PR, progesterone receptor

Sakamoto *et al.*
Figure S2
